# Non-O blood types are associated with a greater risk of large artery atherosclerosis stroke and dysregulation of cholesterol metabolism: an observational study

**DOI:** 10.1186/s12944-024-02199-6

**Published:** 2024-07-04

**Authors:** Lan Gou, Haowen Li, Yingyu Jiang, Yang Liu, Hongqiu Gu, Zhe Xu, Weina Jin, Lanxin Li, Yanfeng Shi, Jie Zhang, Zhenjuan Fang, Xia Meng, Yong Jiang, Hao Li, Yongjun Wang, Si Cheng

**Affiliations:** 1 Department of Neurology, Beijing Tiantan Hospital, Capital Medical University, Beijing, China; 2 China National Clinical Research Center for Neurological Diseases, Beijing Tiantan Hospital, Capital Medical University, Beijing, China; 3 Center of excellence for Omics Research (CORe), Beijing Tiantan Hospital, Capital Medical University, Beijing, China; 4 Changping Laboratory, Beijing, China; 5 Clinical Center for Precision Medicine in Stroke, Capital Medical University, Beijing, China

**Keywords:** Ischaemic stroke, Non-O blood type, Stroke subtype, Cholesterol uptake and efflux, ABO blood type, Large artery atherosclerosis

## Abstract

**Background:**

Previous research on ABO blood types and stroke has been controversial, predominantly suggesting heightened risk of stroke in non-O blood types. Nonetheless, investigations into the correlation and underlying mechanisms between ABO blood groups and stroke subtypes, especially within Chinese cohorts, remain limited.

**Methods:**

The ABO blood types of 9,542 ischaemic stroke (IS) patients were inferred using two *ABO* gene loci (c.261G > del; c.802G > A). The healthy population was derived from the 1000 Genomes Project. Patients were classified by the causative classification system (CCS). Volcano plot and gene ontology (GO) analysis were employed to explore protein differential expression among blood types. Additionally, HT29 and SW480 cell lines with downregulated *ABO* expression were generated to evaluate its impact on cholesterol uptake and efflux.

**Results:**

A greater proportion of stroke patients had non-O blood types (70.46%) than did healthy individuals (61.54%). Notable differences in blood type distributions were observed among stroke subtypes, with non-O blood type patients mainly classified as having large artery atherosclerosis (LAA). Clinical baseline characteristics, such as the low-density lipoprotein cholesterol level, activated partial thromboplastin time and thrombin time, varied significantly among blood types. A volcano plot revealed 17 upregulated and 42 downregulated proteins in the O blood type. GO term analysis indicated that downregulated proteins were primarily associated with lipid metabolism pathways. In vitro experiments revealed that reducing *ABO* gene expression decreased cholesterol uptake and increased cholesterol efflux.

**Conclusions:**

This study revealed that the non-O blood type increased the risk of LAA stroke through cholesterol metabolism.

**Supplementary Information:**

The online version contains supplementary material available at 10.1186/s12944-024-02199-6.

## Background

Stroke has the highest incidence in China and worldwide and is the primary cause of death and disability among Chinese adults. Approximately 82.6% of stroke patients in China suffer from ischaemic stroke (IS). [1, 2]. While ABO blood types have been extensively studied in relation to coronary heart disease (CAD) [3, 4], research on the link to stroke is relatively scarce. Additionally, these findings are controversial [5, 6], predominantly indicating a higher stroke risk for individuals with non-O blood types.

Given the heterogeneity in IS, subtype classification is essential for exploring its underlying aetiological mechanisms. Nonetheless, few studies have examined the associations among ABO blood types and IS subtypes [5]. Most existing research has primarily investigated how the *ABO* gene affects stroke subtypes [7–9]. *ABO* rs505922 has been previously shown to be highly correlated with an increased risk of large artery atherosclerosis (LAA) and cardioembolism (CE) stroke [9].

Previous studies have also suggested that non-O blood types or *ABO* genes may affect coagulation [10], inflammation [11], and lipid metabolism [12]. The mechanisms underlying the effects of the ABO blood type on coagulation factors might be mediated primarily by ABO(H) carbohydrate structures on the surface of both VWF and platelet glycoprotein receptors [13, 14]. The *ABO* gene may influence inflammation by regulating adhesion factor expression, affecting leukocyte attachment to blood vessel walls [11]. O blood type individuals often have lower levels of low-density lipoprotein cholesterol (LDL-C) and total cholesterol (TC) [15]. The loci within the *ABO* gene associated with cholesterol metabolism are in strong linkage disequilibrium with SNP rs8176719, which determines blood type O [16, 17]. However, the precise mechanisms by which the *ABO* gene affects lipid metabolism remain poorly understood, with limited experimental evidence. Previous analyses have certain limitations, including the relatively small number of cases with/without detailed and consistent subtyping information, the lack of experimental evidence, and the limited data available in Chinese populations. This study investigated 9,542 Chinese IS patients with consistent subtyping and rich clinical information, aiming to evaluate how blood types influence IS subtypes and explore the mechanisms through gene ontology (GO) analysis and in vitro experiments.

## Methods

### Study population

Patients were recruited from the Third China National Stroke Registry (CNSR-III), which included 201 sites across 26 regions in China. The registry is a nationwide cohort study for individuals aged 18 years or older who had acute ischaemic stroke (AIS) or transient ischaemic attack (TIA) within a week of symptom onset [18]. All patients provided informed consent. The study was approved by the ethics committees of Beijing Tiantan Hospital (IRB approval number: KY2015-001-01). This article follows the STROBE reporting guidelines.

The CNSR-III cohort included 10,914 patients from the prespecified genetic substudy underwent whole-genome sequencing (WGS) using the BGISEQ-500 platform, following previously described protocols [19]. After passing quality control, 10,241 genetically independent samples were included in further analyses [20]. 11 patients who failed *ABO* gene locus sequencing and 688 patients with TIA were excluded. Overall, the study included 9,542 IS patients, with 2,174 having a history of stroke and 7,368 being first-ever IS patients (Fig. S1). To avoid potential confounding factors that were associated with medication effects among patients with stroke histories, only 7,368 first-ever IS patients were included for further baseline characteristic analyses. The genetic data complied with the Human Genetic Resources Administration of China (HGRAC) regulations. 208 healthy Chinese individuals were identified from the 1000 Genomes Project (https://www.internationalgenome.org/).

### ABO blood type genotyping

The ABO blood type was imputed for genotyped participants using two genetic polymorphic loci of the *ABO* gene on chromosome 9q34.2 that affect amino acid coding. c.261G > del, i.e., rs8176719, was used to infer the deletional O alleles; c.802G > A was used to infer the nondeletional O alleles. If neither of the two gene mutation loci could be detected, it was inferred that the patient had the non-O allele [21]. After extracting the genotype of the two loci for each sample, Shapeit software was used to infer the haplotype and obtain the combination of the two loci for each sample to infer the blood type. The software’s default parameters were used for haplotype inference. Genotypes at the *ABO* gene locus were considered valid when the depth (DP) was ≥ nine and the genotype quality (GQ) was ≥ 20. Heterozygous variants necessitated an allele depth (AD) of ≥ three [20].

### Baseline data collection

The baseline data were collected at each site by trained research coordinators through face-to-face encounters or telephone interviews [18]. Patient demographic information was collected at admission, including age, sex, and ethnicity; traditional cerebrovascular risk factors, including a history of diabetes, dyslipidaemia, hypertension, CAD, atrial fibrillation, current smoking, alcohol abuse (average daily alcohol intake ≥ 20 g), as well as body mass index (BMI); laboratory data, including LDL-C level, TC level, high-density lipoprotein cholesterol (HDL-C) level, TG level, lipoprotein phospholipase A2 (Lp-PLA2) activity and mass, Apo A1 level, Apo B level, lipoprotein(a) (Lp[a]) level, proprotein convertase subtilisin/kexin type 9 (PCSK9) level, cholesterol efflux capacity (CEC), human chitinase 3-like protein 1 (YKL40) level, C-reactive protein (CRP) level, interleukin-6 (IL-6) level, interleukin-1 receptor antagonist (IL-1ra) level, fibrinogen level, D dimer level, activated partial thromboplastin time (APTT), prothrombin time (PT), international normalized ratio (INR), and thrombin time (TT) [18].

A blood pressure reading of ≥ 140/90 mm Hg, antihypertensive medication, or a self-reported history was used to diagnose hypertension. Diabetes mellitus was characterized by a self-reported physician diagnosis, taking hypoglycemic drugs or glucose concentration of 7.0 mmol/L or higher on admission. Dyslipidaemia, atrial fibrillation, and CAD were identified by a self-reported history or diagnosis during hospitalization. The laboratory index detection methods used were detailed in the supplementary materials.

### Aetiological classification

In this study, the causative classification system (CCS) was utilized to categorize stroke patients into five subtypes. CCS is a web-based, evidence-based classification system, which classifies IS into supra-aortic LAA, CE, small artery occlusion (SAO), other causes (OE) and undetermined causes (UE) stroke [22]. Suspected aetiological diagnoses were assessed and determined by a centrally trained expert group specializing in aetiological classification, consisting of three deputy chief physicians or above who used the web-based protocol.

### Proteomics data source

Proteomic analysis was conducted on 164 IS patients without a history of diabetes or heart disease. Blood samples were taken into EDTA tubes (BD Vacutainer EDTA tubes, Franklin Lakes, NJ) and centrifuged for ten minutes at 2–8 °C at 3,000 rpm. The resulting plasma was aliquoted and preserved at -80 °C. The proteins underwent extraction and digestion, followed by analysis with liquid chromatography tandem mass spectrometry (LC-MS/MS) in data-independent acquisition (DIA) mode.

### Analysis of differentially expressed proteins in different blood groups

A volcano plot was constructed by plotting the t test *p* values against the fold change. A p value below 0.05 and a log2-fold change exceeding 1.2 were regarded as statistically significant. GO analysis was carried out in R using the clusterProfiler package, with all detected proteins as the background set. Q-values were calculated using the FDR tool, with a significance threshold set at less than 0.05.

### Cell culture

The HT-29 and SW480 cell lines, obtained from China Infrastructure of Cell Line Resource, were cultured in DMEM/F-12 (Gibco) medium and RPMI 1640 medium (Gibco) with 10% fetal bovine serum (FBS, Gibco), respectively. The cultures were maintained at 37 °C with an atmosphere of 5% CO2 and 95% air.

### Generation of shRNA-knockdown cell lines

ShRNA knockdown lentiviral particles for the *ABO* gene and control were produced by GeneChem (Shanghai, China). The target sequence for the human *ABO* gene was CACTTCGACCTATGATCCTTT. HT-29 and SW480 cells were infected with lentivirus, followed by selection using 2 µg/mL puromycin for a minimum of 7 days prior to conducting further experiments. The infection rate was evaluated through the expression of red fluorescent protein after incubation with lentivirus for 48 h. The interference expression efficiency was detected using real-time PCR.

### Real-time quantitative PCR

RNA was isolated following the manufacturer’s protocol using the TRIzol reagent (Invitrogen). Reverse transcription was performed on 1 µg of RNA using the PrimeScript RT reagent kit with gDNA Eraser (Takara). Real-time PCR was conducted with the Quant Studio 3 qPCR system (ABI) using SYBR Premix Ex TaqII (Takara) and specific primers (ABO-FW, GGGGTTCTGCATGGCTGTTA; ABO-RV, CCTGAACTGCTCGTTGAGGA). The expression values were normalized to those of GAPDH (forward primer, CTATAAATTGAGCCCGCAGCC; reverse primer, GCGCCCAATACGACCAAATC).

### In vitro NBD cholesterol uptake assay

To conduct the NBD cholesterol uptake assay, HT-29 and SW480 cells were seeded in black flat-bottomed 96-well plates. The cells were incubated in complete medium (without phenol red) containing 0.2, 1, 5, or 10 µM NBD-cholesterol (Invitrogen) for 1 h under standard CO2 incubation conditions at 37 °C. Cells were rinsed in PBS three times to remove any NBD cholesterol. Cellular cholesterol was isolated with 0.1% Triton X-100. The fluorescence was quantified using a microplate spectrophotometer (SPARK, Tecan, Austria) at 469 nm for excitation and 537 nm for emission.

### NBD-cholesterol efflux assay

HT-29 and SW480 cells were kept on black flat bottom 96-well plates and exposed to 5 µM NBD-cholesterol in medium without phenol red for a duration of four hours at a temperature of 37 °C. Following incubation, the cells were rinsed thrice with PBS, then placed for 0.5 h in media with 50 mg/L HDL or 50 mg/L ApoA1. The culture medium was collected, and cells were lysed with 0.1% Triton X-100. Fluorescence measurements for the medium and cell lysates were conducted at 469 nm excitation and 537 nm emission. The efflux percentage was computed using the formula: FI (medium) / [FI (medium) + FI (cell lysate)] × 100%.

### Statistical analysis

Baseline characteristics of categorical variables were analysed using Chi-square statistics, while the Wilcoxon rank-sum test was employed for continuous data. Statistics were significant with p values < 0.05.

## Results

### Characteristics of the participants

This study included 9,542 IS patients for analysis (Fig. S1). The distribution of the ABO blood groups in 9,542 IS patients and 208 healthy Chinese individuals was presented in Table S1. Non-O groups were more prevalent among the IS patients than among the healthy group, with a prevalence of 70.46% versus 61.54%, respectively. In both the first-ever IS patients and patients with stroke history, non-O blood type individuals constituted a similar proportion (70.33% vs. 70.88%) but exhibited significant differences in most baseline clinical characteristics and CCS subtype distributions (*P* < 0.001) (Table S2). This indicated that non-O blood groups may influence both stroke onset and recurrence, potentially through different underlying mechanisms.

Among the 9,542 IS patients, 2738 (28.69%) had the LAA subtype, 668 (7.00%) had the CE subtype, 2576 (27.00%) had the SAO subtype, 76 (0.80%) had the OE subtype, and 3484 (36.51%) had the UE subtype. A total of 7,368 out of 9,542 IS patients were first-ever IS patients; among them, 2055 (27.89%) were categorized as the LAA subtype, 502 (6.81%) as the CE subtype, 2060 (27.96%) as the SAO subtype, 59 (0.80%) as the OE subtype, and 2692 (36.54%) as the UE subtype. Overall, among the 9,542 IS patients and the subset of first-ever IS patients, the CCS subtypes showed a similar distribution. Notably, significant disparities in the CCS subtype distribution between the two blood types were observed in both populations, with the non-O blood group exhibiting a notably greater incidence of LAA stroke (Table 1 and S3). This difference also remained significant after a combined comparison with CE and SAO stroke (Tables S4 and S5).

**Table 1:** CCS classifications in O and non-O blood types for IS patients (*N* = 9,542) in CNSR-III

Ischaemic stroke patients (*N* = 9,542)	LAA *N* = 2738	CE *N* = 668	SAO *N* = 2576	OE *N* = 76		UE *N* = 3484	P* (non-O vs. O)
O (n, %)	708 (25.12)	193 (6.85)	806 (28.59)		21 (0.74)	1091(38.70)	< 0.0001
Non-O (n, %)	2030 (30.19)	475 (7.07)	1770 (26.33)		55 (0.82)	2393 (35.59)	

*P: *P* value evaluating the statistical significance of the differences in the distribution of CCS classifications between O and non-O blood type groups.

n: Number of individuals within each blood type group and CCS classification.

%: Percentage of individuals within each CCS classification for each blood type group.

N: Total number of individuals in each CCS classification.

CCS, Causative Classification System; LAA, large artery atherosclerosis; CE, cardioembolism; SAO, small artery occlusion; OE, other causes; UE, undetermined causes.

### Baseline characteristics

To further explore the mediating factors of the blood group on LAA stroke, 32 baseline characteristics were compared between the two blood groups in 7,368 first-ever IS patients (Table 2). These 32 variables included demographic data, 8 risk factors for IS, and 21 biomarkers, including lipid-, inflammation-, and anticoagulation-related biomarkers. Non-O individuals had notably greater LDL-C levels and shorter APTTs and TTs than did individuals in the O blood group. Moreover, the non-O blood types tended to have greater TC levels than the O blood group. There were no significant variations in age, sex, ethnicity, IS risk factors, or inflammation biomarkers between the two blood types. The distribution of blood types O and non-O showed no significant difference between Han and non-Han groups (Table S6).

**Table 2:** Demographics, risk factors, and laboratory results of first-ever ischaemic stroke patients in the CNSR-III cohort

Characteristics	O (*N* = 2186)	Non-O (*N* = 5182)	*P* (O vs. non-O)
**Demographics**			
Age (yr), Median (IQR)	62.0 (53.0–70.0)	62.0 (54.0–69.0)	0.55
Male, n (%)	1500 (68.6)	3581 (69.1)	0.68
Ethnicity (Han nationality), n (%)	2125 (97.2)	5038 (97.2)	0.98
**Risk factors**			
Diabetes, n (%)	729 (33.3)	1766 (34.1)	0.54
Hypertension, n (%)	1674 (76.6)	3862 (74.5)	0.06
Dyslipidaemia, n (%)	844 (38.6)	2033 (39.2)	0.62
Coronary heart disease, n (%)	333 (15.2)	821 (15.8)	0.51
Atrial fibrillation, n (%)	160 (7.3)	356 (6.9)	0.49
Current smoker, n (%)	726 (33.2)	1781 (34.4)	0.34
Heavy drinking, n (%)	333 (15.2)	837 (16.2)	0.32
BMI, median (IQR), kg/m^2^	24.5 (22.6–26.5)	24.5 (22.6–26.7)	0.50
**Laboratory data, median (IQR)**			
LDL-C, mmol/L	2.3 (1.7-3.0)	2.4 (1.8-3.0)	0.03
TC, mmol/L	4.0 (3.3–4.8)	4.0 (3.4–4.8)	0.06
HDL-C, mmol/L	0.9 (0.8–1.1)	0.9 (0.8–1.1)	0.39
TG, mmol/L	1.4 (1.1–1.9)	1.4 (1.0-1.9)	0.51
Lp-PLA2 activity, nmol/min/ml	162.0 (130.1-194.6)	165.2 (130.2–196.0)	0.14
Lp-PLA2 mass, ng/ml	172.8 (125.4-225.8)	177.7 (129.1-226.9)	0.12
Apo A1, g/L	1.2 (1.1–1.4)	1.2 (1.1–1.4)	0.07
Apo B, g/L	0.9 (0.8–1.1)	0.9 (0.8–1.1)	0.12
Lp(a), mg/dL	147.3 (71.7-286.1)	142.1 (71.3-279.4)	0.71
PCSK9, ng/ml	361.7 (286.3-449.1)	362.1 (279.5-453.3)	0.96
CEC, %	21.5 (15.4–27.1)	21.4 (15.4–27.4)	0.62
YKL40, pg/ml	63 203.7 (37 662.0-120 391.3)	62 623.5 (37 263.0-116 853.0)	0.49
Hypersensitive CRP, mg/L	1.7 (0.8–4.5)	1.8 (0.8–4.6)	0.34
IL-1ra, pg/ml	336.8 (249.6-490.2)	341.7 (257.0-493.5)	0.24
IL-6, pg/ml	2.5 (1.5–4.8)	2.7 (1.6-5.0)	0.05
Fibrinogen, mg/dl	378.0 (315.0-445.2)	378.0 (310.8-447.3)	0.82
D dimer, µg/ml	1.1 (0.6–2.1)	1.1 (0.6–2.1)	0.47
APTT, s	30.3 (26.7–34.4)	29.0 (25.7–32.7)	< 0.001
INR	1.0 (0.9-1.0)	1.0 (0.9-1.0)	0.43
PT, s	11.7 (10.9–12.7)	11.7 (10.9–12.6)	0.19
TT, s	16.8 (15.2–18.3)	16.7 (15.0-18.1)	0.03

IQR, interquartile range; BMI, body mass index; LDL-C, low-density lipoprotein cholesterol; TC, total cholesterol; HDL-C, high-density lipoprotein cholesterol; TG, triglycerides; Lp-PLA2, lipoprotein-associated phospholipase A2; Apo, apolipoprotein; Lp(a), lipoprotein(a); PCSK9, proprotein convertase subtilisin/kexin type 9; CEC, cholesterol efflux capacity; YKL40, human chitinase 3-like protein 1; CRP, C-reactive protein; IL-1ra, interleukin-1 receptor antagonist; IL-6, interleukin-6; APTT, activated partial thromboplastin time; INR, international normalized ratio; PT, prothrombin time; TT, thrombin time

### The plasma protein profile variations in the O and non-O blood populations

**Fig. 1:**
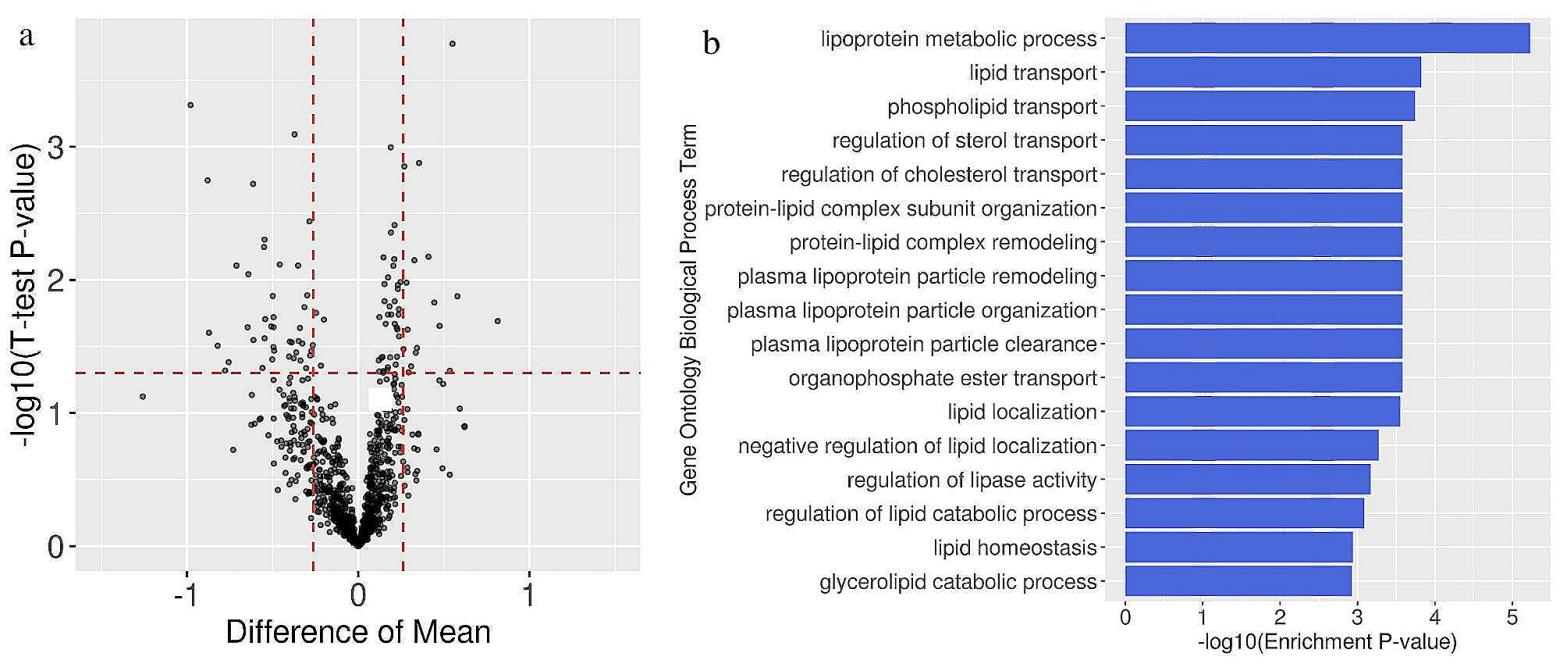
Protein expression analysis in O and non-O blood type patients. (**a**) Volcano plot showing the protein expression differences. X-axis: log-transformed average expression difference. Y-axis: *P* value from t tests. The red line indicates the differential expression threshold. Upper right: upregulated proteins in the O group. Upper left: downregulated proteins in the O group. (**b**) GO biological pathway enrichment analysis of the downregulated proteins in the O group

Among the 974 identified proteins, 17 were upregulated and 42 were downregulated in O type patients relative to the non-O type patients. GO analysis revealed that the enriched pathways were related to lipid metabolism, including lipoprotein metabolic processes, lipid transport, phospholipid transport, regulation of sterol transport, regulation of cholesterol transport, protein-lipid complex subunit organization, protein-lipid complex remodelling, plasma lipoprotein particle remodelling, plasma lipoprotein particle organization, plasma lipoprotein particle clearance, organophosphate ester transport, lipid localization, negative regulation of lipid localization, regulation of lipase activity, regulation of lipid catabolic processes, lipid homeostasis and glycerolipid catabolic processes (Fig. 1).

### Cholesterol Uptake and efflux functionality of HT29 and SW480 cell lines

**Fig. 2:**
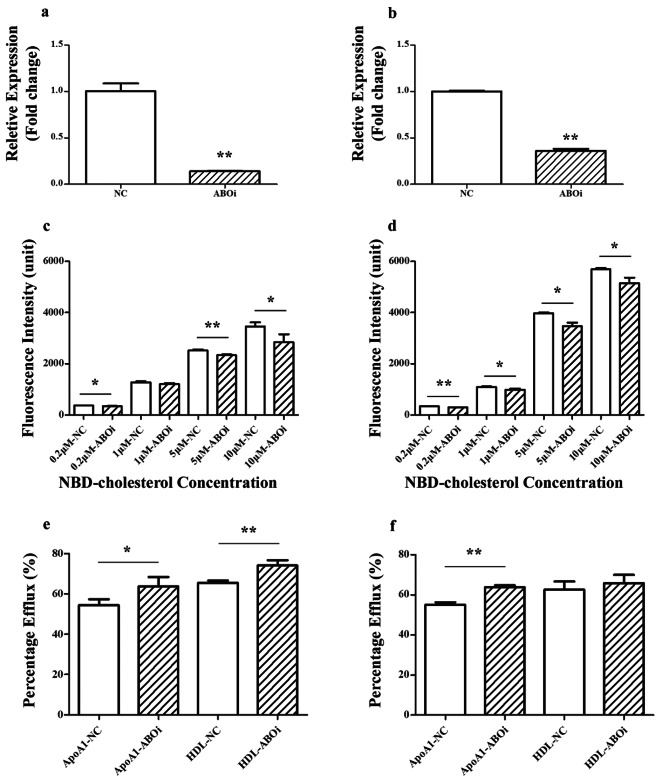
*ABO* gene expression interference can decrease cholesterol uptake and increase cholesterol efflux. *ABO* expression detected in HT29 (**a**) and SW480 (**b**) cells postlentivirus transfection. NBD-cholesterol (0.2, 1, 5, or 10 µM) treatment for 1 h; fluorescence was measured in HT-29 (**c**) and SW480 (**d**) cells. After 4 h of NBD-cholesterol loading, cholesterol efflux was induced by 50 mg/L ApoA1 or HDL for 0.5 h; the percentage of cholesterol efflux was measured in HT-29 (**e**) and SW480 (f) cells. *p < 0.05, **p < 0.01

The expression levels of *ABO* were reduced to 0.14- and 0.36-fold in the *ABO* gene-interfered HT29 (Fig. 2a) and SW480 (Fig. 2b) cells, respectively. After treatment with 0.2, 1, 5, and 10 µM NBD-cholesterol for 1 h, cholesterol uptake was reduced by 9.20%, 5.53%, 7.12%, and 18.04%, respectively, in the *ABO*-interfered HT29 cells (Fig. 2c); in SW480 cells, *ABO* gene downregulation reduced cholesterol uptake by 12.43%, 9.55%, 12.69% and 9.65%, respectively (Fig. 2d). After 4 h of cholesterol loading with 5 µM NBD-cholesterol, cholesterol efflux was induced by 50 mg/L ApoA1 or 50 mg/L HDL for 0.5 h, and the rate of cholesterol efflux in the *ABO* gene-interfered HT29 cells increased by 9.29% and 8.62%, respectively, compared with that in the control group (Fig. 2e); similarly, it was increased by 8.75% and 3.15%, respectively, in the *ABO*-treated SW480 cells (Fig. 2f). Therefore, it can be inferred that downregulation of *ABO* gene expression results in reduced cholesterol uptake and increased cholesterol efflux.

## Discussion

The patients in this study were derived from a large-scale IS cohort in which non-O blood type individuals were more prevalent. The non-O blood group had a notably greater proportion of LAA than the other IS subtypes. Comprehensive phenotypic data, including lipid-related biomarkers, coagulation factors, and inflammatory biomarkers, along with proteomic analysis, provided evidence supporting lipid metabolism as a potential mediating mechanism. Furthermore, cellular experiments using HT29 and SW480 cell lines showed that reducing *ABO* gene expression could decrease cholesterol absorption and increase cholesterol efflux. This study provided crucial evidence highlighting that the non-O blood group was more susceptible to the LAA subtype by regulating cholesterol metabolism.

Several previous observational studies and meta-analyses have shown no associations between ABO blood types and IS [5, 6, 23]. Nonetheless, other evidence suggested an elevated risk of IS in non-O blood type carriers [4, 24, 25]. The IS patients in this study exhibited a greater prevalence of non-O blood types, supporting an elevated risk of IS in individuals with these blood types.

Given the complexity of stroke and its diverse aetiologies, aetiological classification is needed to explore potential pathogenic mechanisms [22]. CCS subtyping is more suitable in multicentre studies and effectively reduces the number of patients classified as “undetermined” compared with Trial of ORG 10,172 in Acute Stroke Treatment (TOAST) subtyping [26].

Multiple investigations consistently proved the link between the *ABO* locus and stroke, with some underscoring its association with the LAA subtype [7, 8, 27]. A meta-analysis involving 12 GWAS studies with 10,307 IS patients and 19,326 healthy individuals, conducted in both Caucasian and South Asian populations, established a robust genome-wide link between the *ABO* locus and the LAA subtype of stroke [27]. This finding was further supported by research in a Chinese Han population, which demonstrated that genetic variations in the *ABO* gene increased the risk of LAA stroke [7]. Additionally, a two-sample Mendelian randomization analysis provided compelling evidence of a causal relationship between the *ABO* gene and the LAA subtype [8]. However, these studies were limited to the impact of SNPs in the *ABO* locus on stroke and lacked further exploration of possible underlying mechanisms.

Several previous studies had attempted to identify the underlying pathways using circulating biomarkers and highlighted coagulation [28], inflammation [11], and lipid metabolism [29] as potential directions. In this study, significant variances in baseline lipid-related and coagulation-related biomarkers among patients with different blood types suggested that O and non-O blood groups influenced LAA stroke through lipid metabolism- or coagulation-related pathways. However, GO analysis revealed that proteins with significant differential expression between the two blood groups were associated with lipid metabolism pathways, without significant involvement of coagulation pathways. Moreover, previous research had indicated the significant effect of the *ABO* gene on cholesterol absorption [17].

Accordingly, both the HT29 and SW480 lines were used to further explore the impact of *ABO* gene expression on cholesterol metabolism. Results from both cell lines consistently revealed that reduced *ABO* gene expression decreased cholesterol absorption and increased efflux processes. The GTEx database confirmed high *ABO* gene expression in the small intestine, the primary site for cholesterol absorption (Fig. S2.). Between O and non-O blood type individuals, details of upregulated and downregulated proteins were provided in supplementary Tables S7 and S8. Downregulated proteins included such as Apo C-I, Apo C-II and Apo C-III. And the downregulated GO terms corresponded to genes such as *APOA1*, *APOC1*, *APOC2*, *APOC3*, and *APOD* (Table S9). Previous research also suggested that interactions, co-expression, genetic interactions, and co-localization of genes (*ABO, APOE, APOA1, AOPA4, APOC2, APOC3*, and 16 others) played significant roles in the cholesterol metabolic pathway [30]. Accordingly, it was hypothesized that the *ABO* gene might have influenced cholesterol binding and transport in the small intestine by affecting the expression of the *Apo* gene cluster, thereby regulating the activity of apolipoproteins [31]. However, further specific molecular mechanisms require more experimental investigation.

### Study strengths and limitations

This study analysed a large national cohort of IS patients, associating ABO blood type with an elevated risk of LAA in non-O individuals. This connection was established through clinical phenotyping, proteomic analysis, and cellular-level experiments. The study combined clinical data with mechanistic validation experiments, unveiling potential mechanisms linking ABO blood type to stroke.

The study also has certain limitations. First, the potential impact of other mechanisms, apart from cholesterol metabolism, on the risk of LAA stroke could not be excluded. Second, the research findings require further animal experiments and validation in other ethnicities.

## Conclusions

In conclusion, the study demonstrated that individuals with blood type O had a lower vulnerability to LAA due to decreased cholesterol absorption and increased efflux, which suggested that cholesterol-lowering drugs, such as ezetimibe, could be beneficial for treating non-O blood type IS patients [32].

### Electronic supplementary material

Below is the link to the electronic supplementary material.

Supplementary Material 1

## Data Availability

Data is provided within the manuscript or supplementary information files.

## Abbreviations

LAA: Large artery atherosclerosis
CE: Cardioembolism
SAO: Small artery occlusion
OE: Other causes
UE: Undetermined causes
CCS: Causative Classification System
TOAST: Trial of ORG 10172 in Acute Stroke Treatment
GO: Gene ontology
IS: Ischaemic stroke
CAD: Coronary heart disease
CNSR-III: Third China National Stroke Registry
AIS: Acute ischaemic stroke
TIA: Transient ischaemic attack
WGS: Whole-genome sequencing
HGRAC: Human Genetic Resources Administration of China
DP: depth
GQ: genotype quality
AD: Allele depth
BMI: Body mass index
LDL-C: Low-density lipoprotein cholesterol
TC: Total cholesterol
HDL-C: High-density lipoprotein cholesterol
TG: Triglycerides
Lp-PLA2: Lipoprotein phospholipase A2
Apo: Apolipoprotein
Lp(a): Lipoprotein(a)
HDL: High-density lipoprotein
PCSK9: Proprotein convertase subtilisin/kexin type 9
CEC: Cholesterol efflux capacity
YKL40: Human chitinase 3-like protein 1
CRP: C-reactive protein
IL-1ra: interleukin-1 receptor antagonist
IL-6: Interleukin-6
APTT: Activated partial thromboplastin time
INR: International normalized ratio
PT: Prothrombin time
TT: Thrombin time
LC-MS/MS: Liquid chromatography tandem mass spectrometry
DIA: Data independent acquisition
FBS: Fetal bovine serum
