# Supplementary material for: Non-O blood types are associated with a greater risk of large artery atherosclerosis stroke and dysregulation of cholesterol metabolism: an observational study
Source: Lipids Health Dis. 2024 Jul 4;23:211. doi: 10.1186/s12944-024-02199-6 (PMC11223365; doi:10.1186/s12944-024-02199-6)
Supplement: Supplementary file 1 — Supplementary Material 1 [file 12944_2024_2199_MOESM1_ESM.docx]

**Supplementary Appendix**

Supplementary to: Non-O blood types are associated with a greater risk of large artery atherosclerosis stroke and dysregulation of cholesterol metabolism: an observational study

[TABLE OF CONTENTS](#_Toc228462579)

[Supplementary methods for baseline data collection 2](#_Toc168357746)

[Figure S1. Flow chart of the Study. 3](#_Toc168357747)

[Figure S2. Multi-tissue expression of the ABO gene from the GTEx database. 4](#_Toc168357748)

[Table S1. ABO blood types in IS patients (CNSR-III) and the healthy Chinese population (1000 Genomes Project). 5](#_Toc168357749)

[Table S2. Baseline characteristics of first-ever and recurrent stroke patients in CNSR-III. 6](#_Toc168357750)

[Table S3. Distribution of CCS aetiological classifications in O and non-O blood types in first-ever IS patients. 7](#_Toc168357751)

[Table S4. Distribution of CCS aetiological classifications (LAA, CE, SAO) in first-ever or recurrent IS patients. 8](#_Toc168357752)

[Table S5. Distribution of CCS aetiological classifications (LAA, CE and SAO) in first-ever or recurrent IS patients. 9](#_Toc168357753)

[Table S6. Distribution of Han and non-Han ethnicity among O and Non-O blood type patients 10](#_Toc168357754)

[Table S7. Upregulation of 17 proteins in O blood type patients compared to non-O blood type patients 11](#_Toc168357758)

[Table S8. Downregulation of 42 proteins in O blood type patients compared to non-O blood type patients. 12](#_Toc168357759)

[Table S9. Downregulated GO terms and their corresponding gene list 16](#_Toc168357760)

## Supplementary methods for baseline data collection

LDL-C, TC, HDL-C, TG, and hsCRP were individually analyzed on the Roche Cobas analyzer. IL-1Ra, IL-6, Lp-PLA2_MASS and PCSK9 were quantified using enzyme-linked immunosorbent assay (ELISA) kits provided by R&D Systems, Inc. located in Minneapolis, MN, USA, with specific catalog numbers as follows: PDRA00B for IL-1Ra, PHS600C for IL-6, DPLG70 for Lp-PLA2_MASS and SPC900 for PCSK9. Apo AI and Apo B were quantified using the Luminex® xMAP® technology with Apomag-62K-06 assay kits provided by Merck Millipore, Inc., located in Darmstadt, Germany. Lp-PLA2-A, D dimer, and Fib levels were assessed using an automated enzyme assay system on an OLYMPUS AU2700 analyzer, utilizing the PLAC test for Lp-PLA2-A provided by Diazyme Laboratories, Inc. in Poway, CA. APTT measures plasma clotting time after adding phospholipids and calcium. PT measures the coagulation time for a mixture of platelet-poor plasma, thromboplastin, and calcium chloride. TT is the time for blood clotting after adding standardized thrombin to plasma. The PT ratio is calculated by dividing the measured plasma PT by the PT of normal human plasma with the same thromboplastin, and INR is determined based on this ratio and the international sensitivity index of the thromboplastin reagent.


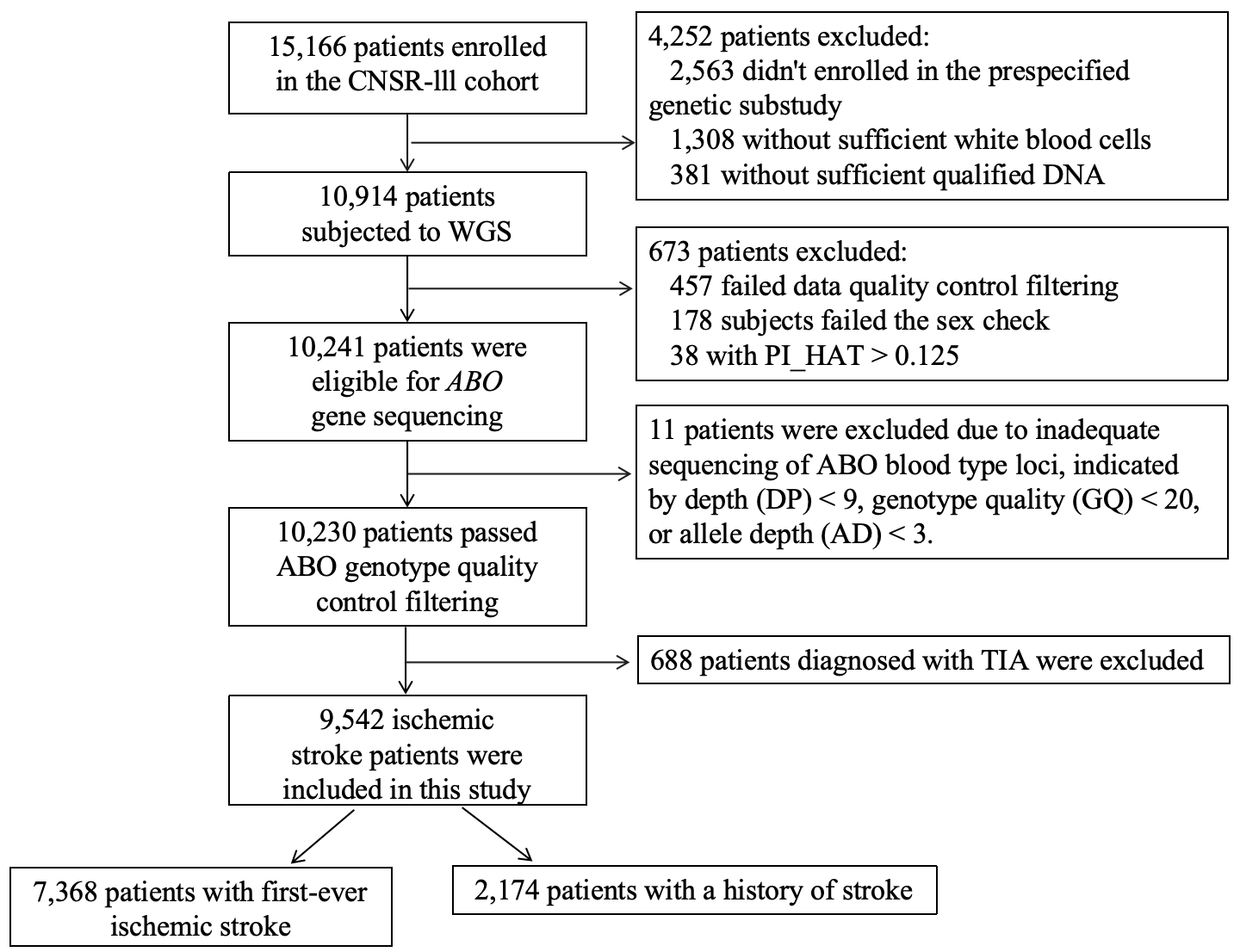


## Figure S1. Flow chart of the Study.

CNSR-Ⅲ: the Third China National Stroke Registry; WGS: whole-genome sequencing; TIA: transient Ischaemic attack. PI_HAT: Proportion IBD (identity by descent).

**
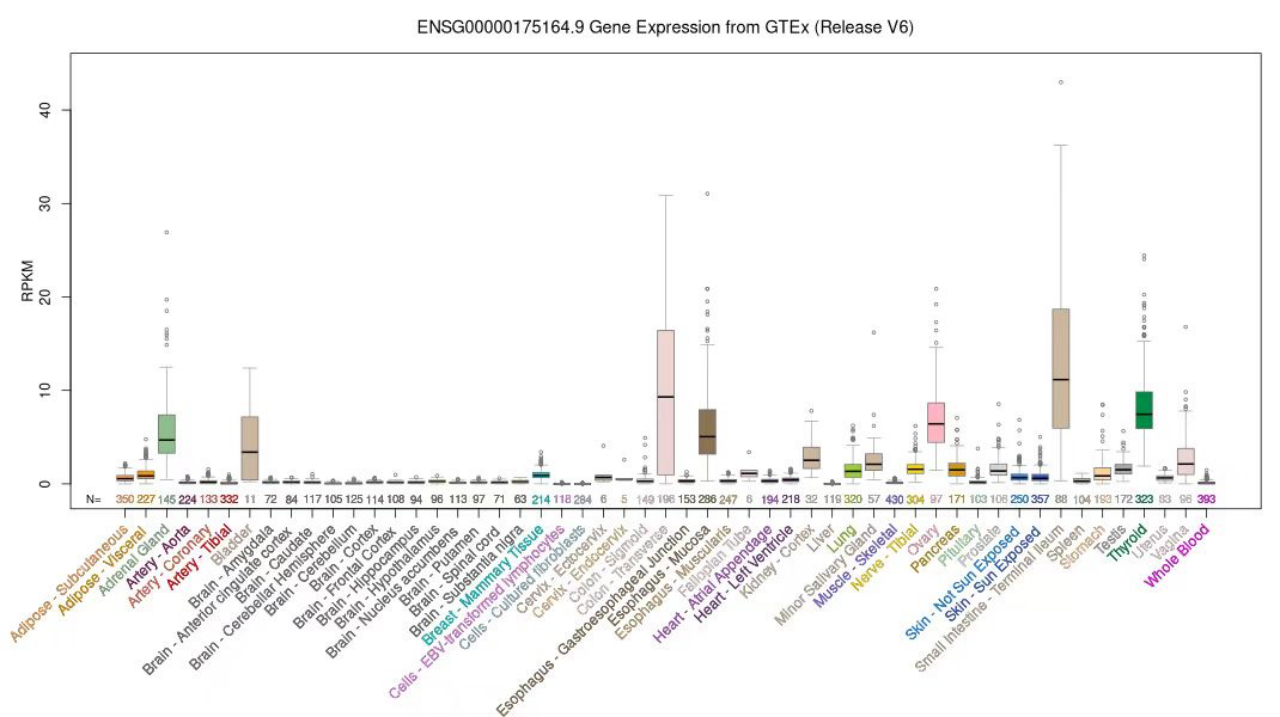
**

## Figure S2. Multi-tissue expression of the ABO gene from the GTEx database.

The *ABO* gene showed high expression in small intestine tissue. Data sourced from the GTEx database. (<https://genome.ucsc.edu/cgi-bin/hgGene?hgg_gene=ENST00000611156.4&hgg_chrom=chr9&hgg_start=133255175&hgg_end=133275214&hgg_type=knownGene&db=hg38>)

## Table S1. ABO blood types in IS patients (CNSR-III) and the healthy Chinese population (1000 Genomes Project).

| **Blood groups** | **CNSR-III**  **(N=9542)** | **The 1000 Genomes Project**  **(CHB and CHS: N=208)** |
| --- | --- | --- |
| O, n (%) | 2819 (29.54) | 80 (38.46) |
| Non-O, n (%) | 6723 (70.46) | 128 (61.54) |

ABO blood types were inferred using two genetic polymorphisms in the *ABO* gene (c.261G>del, i.e., rs8176719; c.802G>A).

“n” represents the number of individuals with different blood types within the cohort, while “%” indicates the percentage of these individuals out of the total cohort population. "N" represents the total number of individuals in different cohorts.

Abbreviations: CNSR-III, The Third China National Stroke Registry-III; CHB, Chinese Han in Beijing; CHS, Chinese Han in Southern China; N/n, number.

## Table S2. Baseline characteristics of first-ever and recurrent stroke patients in CNSR-III.

| **Characteristics** | **First-ever IS patients**  **N = 7368 (77.2%)** | **Patients with stroke history**  **N= 2174 (22.8%)** | ***P*** |
| --- | --- | --- | --- |
| Male, n (%) | 5081 (69.0) | 1525 (70.1) | 0.29 |
| Age (yr), Median (IQR) | 62.0 (53.0-70.0) | 64.0 (58.0-72.0) | <.001 |
| Ethnicity (Han nationality), n (%) | 7163 (97.2) | 2102 (96.7) | 0.20 |
| Current smoker, n (%) | 2507 (34.0) | 623 (28.7) | <.001 |
| Heavy drinker*, n (%) | 1170 (15.9) | 252 (11.6) | <.001 |
| **Medical history, n (%)** | | | |
| Diabetes | 2495 (33.9) | 864 (39.7) | <.001 |
| Hypertension | 5536 (75.1) | 1821 (83.8) | <.001 |
| Dyslipidaemia | 2877 (39.0) | 898 (41.3) | 0.06 |
| Coronary heart disease | 1154 (15.7) | 492 (22.6) | <.001 |
| Atrial fibrillation | 516 (7.0) | 173 (8.0) | 0.13 |
| **Blood type, n (%)** | | | |
| O | 2186 (29.67) | 633 (29.12) | 0.62 |
| Non-O | 5182 (70.33) | 1541 (70.88) |  |
| **CCS subtype** | | | |
| LAA | 2055(27.89) | 683 (31.42) | 0.0005 |
| CE | 502 (6.81) | 166 (7.64) |  |
| SAO | 2060 (27.96) | 516 (23.74) |  |
| OE | 59 (0.80) | 17 (0.78) |  |
| UE | 2692 (36.54) | 792 (36.43) |  |

“n” represents the number of individuals with specific characteristics in different populations, while “%” following “n” indicates the percentage of individuals with these characteristics in their respective populations.

"N" represents the total number of individuals in the sample, while “%” following “N” indicates the percentage of individuals in that specific group within the total study population.

* Heavy drinker defined as alcohol consumption ≥20g/per day

Abbreviation: IQR, interquartile range; CCS, Causative Classification System; LAA, large artery atherosclerosis; CE, cardioembolism; SAO, small artery occlusion; OE, other causes; UE, undetermined causes

## Table S3. Distribution of CCS aetiological classifications in O and non-O blood types in first-ever IS patients.

| **first-ever Ischaemic stroke patients (N=7,368)** | LAA  N=2055 | CE  N=502 | SAO  N=2060 | OE  N=59 | UE  N=2692 | ***P****  **(non-O vs. O)** |
| --- | --- | --- | --- | --- | --- | --- |
| O (n, %) | 526 (24.06) | 151 (6.91) | 648 (29.64) | 18 (0.82) | 843 (38.56) | 0.0001 |
| Non-O (n, %) | 1529 (29.51) | 351 (6.77) | 1412 (27.25) | 41 (0.79) | 1849 (35.68) |  |

**P*: *P* value evaluating the statistical significance of the differences in the distribution of CCS classifications between O and non-O blood type groups.

n: Number of individuals within each blood type group and CCS classification.

%: Percentage of individuals within each CCS classification for each blood type group.

N: Total number of individuals in each CCS classification.

Abbreviations: CCS, Causative Classification System; LAA, large artery atherosclerosis; CE, cardioembolism; SAO, small artery occlusion; OE, other causes; UE, undetermined causes

## Table S4. Distribution of CCS aetiological classifications (LAA, CE, SAO) in first-ever or recurrent IS patients.

| **First-ever Ischaemic stroke patients (N=7,368)** | LAA  N=2055 | CE  N=502 | SAO  N=2060 | ***P****  **(non-O vs. O)** |
| --- | --- | --- | --- | --- |
| O (n,%) | 526 (39.70) | 151 (11.40) | 648 (48.91) | 0.0001 |
| Non-O (n,%) | 1529 (46.45) | 351 (10.66) | 1412 (42.89) |  |
| **Ischaemic stroke patients (N=9,542)** | LAA  N=2738 | CE  N=668 | SAO  N=2576 | ***P****  **(non-O vs. O)** |
| O (n,%) | 708 (41.48) | 193 (11.31) | 806 (47.22) | <0.0001 |
| Non-O (n,%) | 2030 (47.49) | 475 (11.11) | 1170 (41.40) |  |

**P*: *P* value evaluating the statistical significance of the differences in the distribution of CCS classifications between O and non-O blood type groups.

n: Number of individuals within each blood type group and CCS classification.

%: Percentage of individuals within each CCS classification for each blood type group.

N: Total number of individuals in each CCS classification.

Abbreviations: CCS, Causative Classification System; LAA, large artery atherosclerosis; CE, cardioembolism; SAO, small artery occlusion

## Table S5. Distribution of CCS aetiological classifications (LAA, CE and SAO) in first-ever or recurrent IS patients.

| **first-ever ischaemic stroke patients (N=7,368)** | LAA  N=2055 | CE and SAO  N=2562 | ***P****  **(non-O vs. O)** |
| --- | --- | --- | --- |
| O (n,%) | 526 (39.70) | 799 (60.30) | <.0001 |
| Non-O (n,%) | 1529 (46.45) | 1763 (53.55) |  |
| **Ischaemic stroke patients (N=9,542)** | LAA  N=2738 | CE and SAO  N=3244 | ***P****  **(non-O vs. O)** |
| O (n,%) | 708 (41.48) | 999 (58.52) | <.0001 |
| Non-O (n,%) | 2030 (47.49) | 2245 (52.51) |  |

**P*: *P* value evaluating the statistical significance of the differences in the distribution of CCS classifications between O and non-O blood type groups.

n: Number of individuals within each blood type group and CCS classification.

%: Percentage of individuals within each CCS classification for each blood type group.

N: Total number of individuals in each CCS classification.

Abbreviations: CCS, Causative Classification System; LAA, large artery atherosclerosis; CE, cardioembolism; SAO, small artery occlusion

## Table S6. Distribution of Han and non-Han ethnicity among O and non-O blood type patients

| **Blood groups** | **Han Ethnicity**  **(N=7163)** | **non-Han ethnicity**  **(N=205)** | ***P****  **(non-O vs. O)** |
| --- | --- | --- | --- |
| O, n (%) | 2125 (97.21) | 61 (2.79) | 0.9779 |
| Non-O, n (%) | 5038 (97.22) | 144 (2.78) |  |

*The *P* value evaluated the ethnic distribution differences between O and non-O blood type patients.

“n” denoted the number of individuals in each blood group, and “%” represented the proportion of each ethnicity within the O and non-O blood groups.

“N” represented the total number of individuals in each ethnic group.

## Table S7. Upregulation of 17 proteins in O blood type patients compared to non-O blood type patients

| UniProt ID | Entrez ID | Symbol | Description | Call Rate | Mean_rs817671O | Mean_rs8176719nonO | Difference | Fold Change | Direction | t-test *P* value | t-test FDR |
| --- | --- | --- | --- | --- | --- | --- | --- | --- | --- | --- | --- |
| P48960 | 976 | ADGRE5 | CD97 antigen | 0.706806283 | 11.18017645 | 10.73545313 | 0.444723323 | 1.361053072 | UP | 0.014812982 | 0.411323113 |
| P55268 | 3913 | LAMB2 | Laminin subunit beta-2 | 0.502617801 | 11.5934939 | 11.18200824 | 0.411485659 | 1.330054774 | UP | 0.006704321 | 0.363958772 |
| P55287 | 1009 | CDH11 | Cadherin-11 | 0.837696335 | 12.11615194 | 11.82137151 | 0.294780424 | 1.226698262 | UP | 0.049556038 | 0.499888109 |
| P09104 | 2026 | ENO2 | Gamma-enolase | 0.586387435 | 12.16861039 | 11.693036 | 0.475574389 | 1.390471707 | UP | 0.022169908 | 0.411323113 |
| P00492 | 3251 | HPRT1 | Hypoxanthine-guanine phosphoribosyl transferase | 0.816753927 | 12.25880848 | 11.96994515 | 0.288863327 | 1.221677362 | UP | 0.023648967 | 0.411323113 |
| P23381 | 7453 | WARS | Tryptophan--tRNA ligase, cytoplasmic | 0.827225131 | 12.13687263 | 11.79353619 | 0.343336437 | 1.268687223 | UP | 0.032453524 | 0.464849008 |
| Q9HBR0 | 124565 | SLC38A10 | Putative sodium-coupled neutral amino acid transporter 10 | 0.910994764 | 11.95434829 | 11.62599588 | 0.328352415 | 1.255578661 | UP | 0.007127181 | 0.363958772 |
| P26572 | 4245 | MGAT1 | Alpha-1,3-mannosyl-glycoprotein 2-beta-N-acetylglucosaminyltransferase | 0.623036649 | 12.55550416 | 11.97629617 | 0.579207987 | 1.494028829 | UP | 0.01327391 | 0.411323113 |
| P15291 | 2683 | B4GALT1 | Beta-1,4-galactosyltransferase 1 | 0.952879581 | 12.57280224 | 12.26363651 | 0.309165725 | 1.238991015 | UP | 0.044632858 | 0.498085285 |
| Q9HBW9 | 64123 | ADGRL4 | Adhesion G protein-coupled receptor L4 | 0.984293194 | 14.04699532 | 13.78203576 | 0.264959554 | 1.201602362 | UP | 0.033128169 | 0.46763531 |
| Q9H4A9 | 64174 | DPEP2 | Dipeptidase 2 | 1 | 13.6903623 | 13.42133615 | 0.269026152 | 1.204994158 | UP | 0.001403508 | 0.22783619 |
| P61106 | 51552 | RAB14 | Ras-related protein Rab-14 | 0.628272251 | 13.61879709 | 13.08352844 | 0.535268652 | 1.449211995 | UP | 0.048080218 | 0.499348254 |
| P18428 | 3929 | LBP | Lipopolysaccharide-binding protein | 1 | 18.19150268 | 17.85442278 | 0.337079901 | 1.263197219 | UP | 0.035254784 | 0.470385753 |
| P04278 | 6462 | SHBG | Sex hormone-binding globulin | 1 | 18.56344949 | 18.01249864 | 0.550950851 | 1.465050963 | UP | 0.000168467 | 0.16408667 |
| Q06033 | 3699 | ITIH3 | Inter-alpha-trypsin inhibitor heavy chain H3 | 1 | 20.57244885 | 20.29028419 | 0.282164654 | 1.216018059 | UP | 0.0104982 | 0.394259438 |
| P02748 | 735 | C9 | Complement component C9 | 1 | 21.86616251 | 21.51053237 | 0.35563014 | 1.279544338 | UP | 0.001325809 | 0.22783619 |
| P27348 | 10971 | YWHAQ | 14-3-3 protein theta | 0.795811518 | 13.2687107 | 12.45371952 | 0.814991183 | 1.7592874 | UP | 0.02042225 | 0.411323113 |

## Table S8. Downregulation of 42 proteins in O blood type patients compared to non-O blood type patients.

| UniProt ID | Entrez ID | Symbol | Description | Call Rate | Mean_rs817671O | Mean_rs8176719nonO | Difference | Fold Change | Direction | t-test *P* value | t-test FDR |
| --- | --- | --- | --- | --- | --- | --- | --- | --- | --- | --- | --- |
| P16233 | 5406 | PNLIP | Pancreatic triacylglycerol lipase | 0.811518325 | 11.21212609 | 11.57408966 | -0.361963564 | 0.778104828 | DOWN | 0.03504453 | 0.470385753 |
| Q14314 | 10875 | FGL2 | Fibroleukin | 0.994764398 | 12.89474041 | 13.17523113 | -0.280490713 | 0.823310932 | DOWN | 0.037025204 | 0.471277925 |
| P05121 | 5054 | SERPINE1 | Plasminogen activator inhibitor 1 | 0.952879581 | 12.7807298 | 13.12082967 | -0.340099868 | 0.789986624 | DOWN | 0.022953147 | 0.411323113 |
| A0A0C4DH68 | -- | -- | Immunoglobulin kappa variable 2-24 | 0.916230366 | 13.65968152 | 14.30084012 | -0.641158603 | 0.641197807 | DOWN | 0.009064304 | 0.394259438 |
| Q86YZ3 | 388697 | HRNR | Hornerin | 1 | 12.04581976 | 12.55379242 | -0.507972657 | 0.703209927 | DOWN | 0.022363951 | 0.411323113 |
| P04908 | 30,128,335 | HIST1H2AE,  HIST1H2AB | Histone H2A type 1-B/E; Histone H2A type 1; Histone H2A type 1-D; Histone H2A type 3; Histone H2A type 1-C; Histone H2A type 1-H; Histone H2A type 1-J; Histone H2A.J | 0.97382199 | 11.99498247 | 12.55414272 | -0.559160256 | 0.678697096 | DOWN | 0.045913536 | 0.498085285 |
| Q9H8L6 | 79812 | MMRN2 | Multimerin-2 | 0.706806283 | 12.25598388 | 13.23497536 | -0.978991483 | 0.507334268 | DOWN | 0.000486025 | 0.22783619 |
| P58335 | 118429 | ANTXR2 | Anthrax toxin receptor 2 | 0.879581152 | 12.94277229 | 13.29104131 | -0.34826902 | 0.785526026 | DOWN | 0.028799729 | 0.456976696 |
| P08294 | 6649 | SOD3 | Extracellular superoxide dismutase [Cu-Zn] | 1 | 13.47125166 | 13.74580498 | -0.274553322 | 0.826706238 | DOWN | 0.034241059 | 0.469729453 |
| Q9UBX5 | 10516 | FBLN5 | Fibulin-5 | 0.963350785 | 14.1751508 | 14.47299887 | -0.297848067 | 0.813464861 | DOWN | 0.013063213 | 0.411323113 |
| P49407 | 408 | ARRB1 | Beta-arrestin-1 | 1 | 15.00476762 | 15.38081591 | -0.376048292 | 0.770545315 | DOWN | 0.038708659 | 0.471277925 |
| O95236 | 80833 | APOL3 | Apolipoprotein L3 | 0.989528796 | 14.20274946 | 14.8164546 | -0.613705139 | 0.65351618 | DOWN | 0.0019023 | 0.231604994 |
| Q9BY67 | 23705 | CADM1 | Cell adhesion molecule 1 | 0.984293194 | 13.60099691 | 13.95196199 | -0.350965077 | 0.784059433 | DOWN | 0.007803523 | 0.363958772 |
| P01833 | 5284 | PIGR | Polymeric immunoglobulin receptor | 0.952879581 | 12.0781691 | 12.62825849 | -0.550089392 | 0.682977808 | DOWN | 0.005662654 | 0.363958772 |
| Q13085 | 31 | ACACA | Acetyl-CoA carboxylase 1 | 1 | 16.46278799 | 16.77669036 | -0.313902365 | 0.804462811 | DOWN | 0.016015261 | 0.411323113 |
| Q6S8J3 | 445582 | POTEE | POTE ankyrin domain family member E | 0.979057592 | 14.29953442 | 15.01092418 | -0.711389762 | 0.610731532 | DOWN | 0.007806738 | 0.363958772 |
| P14151 | 6402 | SELL | L-selectin | 1 | 16.51657561 | 16.82074841 | -0.304172806 | 0.809906457 | DOWN | 0.046024308 | 0.498085285 |
| O75821 | 8666 | EIF3G | Eukaryotic translation initiation factor 3 subunit G | 1 | 16.69741204 | 17.06924391 | -0.371831873 | 0.772800604 | DOWN | 0.000806853 | 0.22783619 |
| P13473 | 3920 | LAMP2 | Lysosome-associated membrane glycoprotein 2 | 1 | 17.16710673 | 17.43273065 | -0.265623923 | 0.831838911 | DOWN | 0.030910308 | 0.461052479 |
| P49908 | 6414 | SELENOP | Selenoprotein P | 1 | 17.82528877 | 18.10984074 | -0.284551974 | 0.820996528 | DOWN | 0.003631675 | 0.363958772 |
| P51149 | 7879 | RAB7A | Ras-related protein Rab-7a | 0.979057592 | 16.0851587 | 16.58399283 | -0.498834123 | 0.707678442 | DOWN | 0.013211615 | 0.411323113 |
| A0A0C4DH31 | -- | -- | Immunoglobulin heavy variable 1-18; Immunoglobulin heavy variable 1-2 | 0.994764398 | 17.49207941 | 18.13811927 | -0.646039861 | 0.639032025 | DOWN | 0.022686977 | 0.411323113 |
| P13645 | 3858 | KRT10 | Keratin, type I cytoskeletal 10 | 1 | 18.36592706 | 18.86767354 | -0.501746481 | 0.706251298 | DOWN | 0.039596576 | 0.473874659 |
| O14791 | 8542 | APOL1 | Apolipoprotein L1 | 1 | 17.84033528 | 18.16723751 | -0.326902224 | 0.797246507 | DOWN | 0.030049353 | 0.457313595 |
| Q08380 | 3959 | LGALS3BP | Galectin-3-binding protein | 1 | 18.96218763 | 19.4531361 | -0.490948478 | 0.711557142 | DOWN | 0.034009275 | 0.469729453 |
| P19652 | 5005 | ORM2 | Alpha-1-acid glycoprotein 2 | 1 | 18.17573409 | 18.7193315 | -0.543597402 | 0.686058069 | DOWN | 0.019715292 | 0.411323113 |
| P0DOX2 | -- | -- | Immunoglobulin alpha-2 heavy chain | 1 | 18.80377957 | 19.29844751 | -0.494667942 | 0.709725013 | DOWN | 0.022648868 | 0.411323113 |
| P61221 | 6059 | ABCE1 | ATP-binding cassette sub-family E member 1 | 0.994764398 | 17.54619026 | 18.42643601 | -0.880245748 | 0.543274882 | DOWN | 0.001789373 | 0.231604994 |
| P29400 | 1287 | COL4A5 | Collagen alpha-5 (IV) chain | 1 | 19.62822541 | 20.0324294 | -0.404203993 | 0.755653109 | DOWN | 0.037620352 | 0.471277925 |
| P20851 | 725 | C4BPB | C4b-binding protein beta chain | 1 | 19.34937669 | 19.68708593 | -0.337709241 | 0.791296762 | DOWN | 0.040381516 | 0.473874659 |
| Q8NG11 | 81619 | TSPAN14 | Tetraspanin-14 | 1 | 19.41989373 | 20.29181656 | -0.871922831 | 0.546418096 | DOWN | 0.024930017 | 0.42599713 |
| P02654 | 341 | APOC1 | Apolipoprotein C-I | 1 | 20.13489291 | 20.53418445 | -0.399291541 | 0.758230533 | DOWN | 0.029112461 | 0.456976696 |
| P02655 | 344 | APOC2 | Apolipoprotein C-II | 1 | 20.82675971 | 21.37381107 | -0.547051356 | 0.684417543 | DOWN | 0.004978948 | 0.363958772 |
| P05090 | 347 | APOD | Apolipoprotein D | 1 | 21.98483584 | 22.53183797 | -0.547002136 | 0.684440893 | DOWN | 0.027449725 | 0.453153085 |
| P02656 | 345 | APOC3 | Apolipoprotein C-III | 1 | 22.96916725 | 23.42763627 | -0.458469019 | 0.727758142 | DOWN | 0.007669908 | 0.363958772 |
| P02652 | 336 | APOA2 | Apolipoprotein A-II | 1 | 23.82797175 | 24.32220179 | -0.49423004 | 0.709940469 | DOWN | 0.019083834 | 0.411323113 |
| P02647 | 335 | APOA1 | Apolipoprotein A-I | 1 | 24.87947235 | 25.63577373 | -0.756301382 | 0.59201212 | DOWN | 0.041479121 | 0.480960281 |
| O94919 | 23052 | ENDOD1 | Endonuclease domain-containing 1 protein | 0.670157068 | 11.73867244 | 12.12737485 | -0.388702412 | 0.763816287 | DOWN | 0.029558041 | 0.456976696 |
| P15289 | 410 | ARSA | Arylsulfatase A | 0.534031414 | 13.06982999 | 13.56492354 | -0.495093544 | 0.709515672 | DOWN | 0.031988862 | 0.464849008 |
| P20930 | 2312 | FLG | Filaggrin | 0.748691099 | 12.72116453 | 13.4986013 | -0.777436767 | 0.583402403 | DOWN | 0.047969005 | 0.499348254 |
| P31025 | 3933 | LCN1 | Lipocalin-1; Putative lipocalin 1-like protein 1 | 0.586387435 | 11.34646203 | 12.16709695 | -0.820634917 | 0.566192712 | DOWN | 0.031241749 | 0.461052479 |
| P50990 | 10694 | CCT8 | T-complex protein 1 subunit theta | 0.963350785 | 16.20410409 | 16.81632479 | -0.612220694 | 0.654188955 | DOWN | 0.028290423 | 0.456976696 |

## Table S9. Downregulated GO terms and their corresponding gene list

| ID | Description | GeneRatio | Bg  Ratio | *P*  value | *P*  adjust | Q  value | Gene ID | Count |
| --- | --- | --- | --- | --- | --- | --- | --- | --- |
| GO:0042157 | lipoprotein metabolic process | 7/40 | 19/921 | 5.86E-06 | 0.005441288 | 0.005209777 | APOL3/APOL1/APOC1/APOD/APOC3/APOA2/APOA1 | 7 |
| GO:0006869 | lipid transport | 9/40 | 50/921 | 0.000149142 | 0.019909736 | 0.019062633 | PNLIP/APOL3/APOL1/APOC1/APOC2/APOD/APOC3/APOA2/APOA1 | 9 |
| GO:0015914 | phospholipid transport | 5/40 | 14/921 | 0.000180234 | 0.019909736 | 0.019062633 | APOC1/APOC2/APOC3/APOA2/APOA1 | 5 |
| GO:0015748 | organophosphate ester transport | 5/40 | 15/921 | 0.000261746 | 0.019909736 | 0.019062633 | APOC1/APOC2/APOC3/APOA2/APOA1 | 5 |
| GO:0032371 | regulation of sterol transport | 5/40 | 15/921 | 0.000261746 | 0.019909736 | 0.019062633 | APOC1/APOC2/APOC3/APOA2/APOA1 | 5 |
| GO:0032374 | regulation of cholesterol transport | 5/40 | 15/921 | 0.000261746 | 0.019909736 | 0.019062633 | APOC1/APOC2/APOC3/APOA2/APOA1 | 5 |
| GO:0034367 | protein-containing complex remodeling | 5/40 | 15/921 | 0.000261746 | 0.019909736 | 0.019062633 | APOC1/APOC2/APOC3/APOA2/APOA1 | 5 |
| GO:0034368 | protein-lipid complex remodeling | 5/40 | 15/921 | 0.000261746 | 0.019909736 | 0.019062633 | APOC1/APOC2/APOC3/APOA2/APOA1 | 5 |
| GO:0034369 | plasma lipoprotein particle remodeling | 5/40 | 15/921 | 0.000261746 | 0.019909736 | 0.019062633 | APOC1/APOC2/APOC3/APOA2/APOA1 | 5 |
| GO:0034381 | plasma lipoprotein particle clearance | 5/40 | 15/921 | 0.000261746 | 0.019909736 | 0.019062633 | APOC1/APOC2/APOC3/APOA2/APOA1 | 5 |
| GO:0071825 | protein-lipid complex subunit organization | 5/40 | 15/921 | 0.000261746 | 0.019909736 | 0.019062633 | APOC1/APOC2/APOC3/APOA2/APOA1 | 5 |
| GO:0071827 | plasma lipoprotein particle organization | 5/40 | 15/921 | 0.000261746 | 0.019909736 | 0.019062633 | APOC1/APOC2/APOC3/APOA2/APOA1 | 5 |
| GO:0010876 | lipid localization | 9/40 | 54/921 | 0.000278608 | 0.019909736 | 0.019062633 | PNLIP/APOL3/APOL1/APOC1/APOC2/APOD/APOC3/APOA2/APOA1 | 9 |
| GO:0048259 | regulation of receptor-mediated endocytosis | 5/40 | 17/921 | 0.000505573 | 0.026021542 | 0.024914399 | SERPINE1/ARRB1/APOC1/APOC2/APOC3 | 5 |
| GO:1905953 | negative regulation of lipid localization | 4/40 | 10/921 | 0.000532195 | 0.026021542 | 0.024914399 | APOC1/APOC2/APOC3/APOA2 | 4 |
| GO:0060191 | regulation of lipase activity | 5/40 | 18/921 | 0.000677751 | 0.031481528 | 0.030142078 | PNLIP/APOC1/APOC2/APOC3/APOA2 | 5 |
| GO:0050994 | regulation of lipid catabolic process | 4/40 | 11/921 | 0.000810117 | 0.035828859 | 0.034304443 | APOC1/APOC2/APOC3/APOA2 | 4 |
| GO:0055088 | lipid homeostasis | 5/40 | 20/921 | 0.001149619 | 0.045565224 | 0.043626553 | ACACA/APOC2/APOC3/APOA2/APOA1 | 5 |
| GO:0046503 | glycerolipid catabolic process | 4/40 | 12/921 | 0.001177142 | 0.045565224 | 0.043626553 | APOC1/APOC2/APOC3/APOA2 | 4 |
